# Public awareness of dementia: A study in Botucatu, a medium-sized city in the State of São Paulo, Brazil

**DOI:** 10.1590/S1980-57642009DN20300005

**Published:** 2008

**Authors:** Arthur Oscar Schelp, Andrea Bruno Nieri, Pedro Tadao Hamamoto Filho, Alessandra Martins Bales, Cristiane Lara Mendes-Chiloff

**Affiliations:** 1 Neurologist, Department of Neurology, Psychology and Psychiatry, Botucatu Medical School, São Paulo State University.; 2 Student, 5th year, Botucatu Medical School, São Paulo State University; 3 Psychologist, Botucatu Medical School, São Paulo State University.

**Keywords:** dementia, Alzheimer’s disease, elderly population, public awareness

## Abstract

Dementia is a progressive and debilitating disease affecting an increasing number of people worldwide. Despite its importance, only a few studies have examined public awareness of dementia. We present a study of the public awareness of dementia in Botucatu, São Paulo, Brazil.

**Methods:**

A sample of 73 individuals answered a questionnaire approved by the Medical Ethics Research Committee inquiring about the characteristics of healthy old-aged and demented individuals.

**Results:**

Those interviewed believed that dementia is characterized by prevalent memory impairment (41%) and behavioral changes (32.9%) with onset in the 60’s or older (42.5%) and upon suspecting dementia, only a few would seek specialized medical help.

**Discussion:**

Better understanding of public awareness of dementia provides the clue to more effective health and social policies in order to achieve a higher rate of early diagnosis and thereby possibly decreasing patient, family and caregiver distress.

## Abstract (pt)

Demência é uma síndrome progressiva e inabilitante que afeta um número crescente de pessoas ao redor do mundo. A despeito de sua importância, poucos estudos tem examinado a conhecimento público sobre demência.

**Objetivo:**

Apresentar um estudo sobre o conhecimento sobre demência em Botucatu, São Paulo, Brasil.

**Métodos:**

Uma amostra de 73 indivíduos responderam a um questionário, aprovado pelo Comitê de Ética em Pesquisa Médica, a respeito das características de indivíduos idosos sadios e pessoas com demência. Resultados: Os sujeitos entrevistados acreditavam que a demência se caracteriza por uma prevalente perda de memória (41%) e alterações comportamentais (32.9%) com início aos 60 anos ou mais (42.5%) e, e no caso de suspeita de demência, somente poucos procurariam ajuda médica especializada.

**Discussão:**

Melhor compreensão do conhecimento público sobre demência fornece pistas para políticas sociais e de saúde mais efetivas a fim de atingir um índice mais alto de diagnóstico precoce e possivelmente diminuir o desgaste do paciente, da família e dos cuidadores.

Alzheimer’s Disease International is an association created to support Alzheimer associations throughout the world, and works to increase the awareness of dementia among the general community and make dementia a public health priority. Nevertheless, only a few studies have been published on the level of perception of dementia in developing countries.^1^ Poor awareness leads to inappropriate recognition and management of dementia in health services, stigmatization of patients and lack of efficient family support (i.e. caregivers).^2^ Cognitive deficits in dementia are characterized by many symptoms, including memory dysfunction, language disorders, impairment of praxis and loss of executive functions.^3^ Recognizing the most frequent signs in a patient with dementia is a major part of training programs for potential caregivers. Lack of information leads to wrong expectations regarding possibilities and limitations of life in old age as well as inappropriate medical and social support for demented patients.

The purpose of this investigation was to evaluate expectancy from the healthy old-aged and scope of dementia, as well as to ascertain which professionals have first contact with cases of suspected dementia.

## Methods

Seventy-three individuals from 40 to 85 years’ old (37 male and 36 female) answered a questionnaire approved by the Medical Ethics Research Committee of Botucatu Medical School – São Paulo State University (Table 1).

**Table 1:** The questionnaire applied.

Questionnaire about dementia			
Age:	years	Gender:	Origin:
1. In your opinion, what happens to one's behavior when he/she gets older ?			
2. Can a person with dementia (sometimes called Alzheimer's disease) stay at home alone ?			
3. At what age does dementia begins ?			
4. What do you expect from someone with dementia ?			
5. Where would you go or what kind of help would you look for it someone in your family shows signs of dementia ?			
6. Do you believe that someone with dementia can have feeding difficulties ?			
7. Wich of the following behaviors would you associate with dementia ?			
a) Forgetting obligations			
b) Forget the next commitment			
c) Unable to perform common tasks (e.g.: cooking, going to the bank, dressing up...)			
d) Feeding problems			
e) Changing names and parenteral status			
f) Unable to comprehend related facts and objects			

The questionnaire was applied twice within different settings. First, at the III Elderly Health Meeting (2007, August), which took place in Jardim Cristina, a neighborhood with a young,, mostly Caucasian population broad access to health and other social services, declining criminality and growing economy, and second, at a public downtown square in Botucatu, São Paulo (2008, April). Most interviewees in the second sample were of mixed ethnicity (Caucasian with African or Indian origins) and lower incomes and educational level.

A broad spectrum of answers was obtained because there were no set items to choose from on the questionnaire. Accordingly, we were able to record spontaneous reply, although this may have inadvertently led to bias in the interpretation. Answers were grouped into:

1 – behavioral/mood changes,
2 – memory impairment,
3 – “movement disorders” (in a general sense) for subsequent data analysis.

## Results

Although there was a socio-economic discrepancy between the two studied populations, the distribution of answers proved similar.

**Graph 2:**
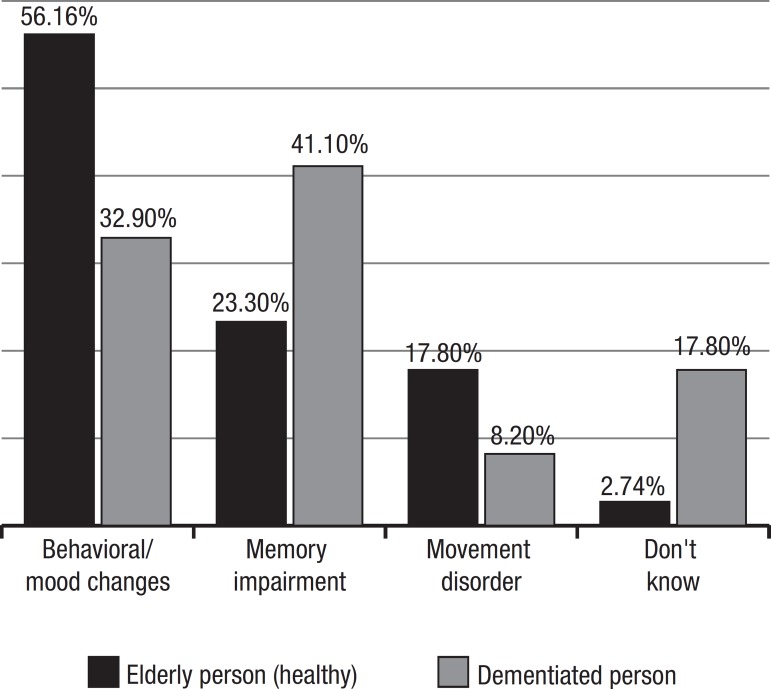
Expected changes from the elderly × characteristics of the demented.

The expected age for the onset of dementia ranged from 30 to 100 years’ old. Most of those interviewed answered 60 years or older (42.5%), 23.3% from 40 to 59, 6.8% from 30 to 39, 20.5% said that symptoms may first appear at any age and 6.8% gave no answer (Graph 2).

**Graph 3:**
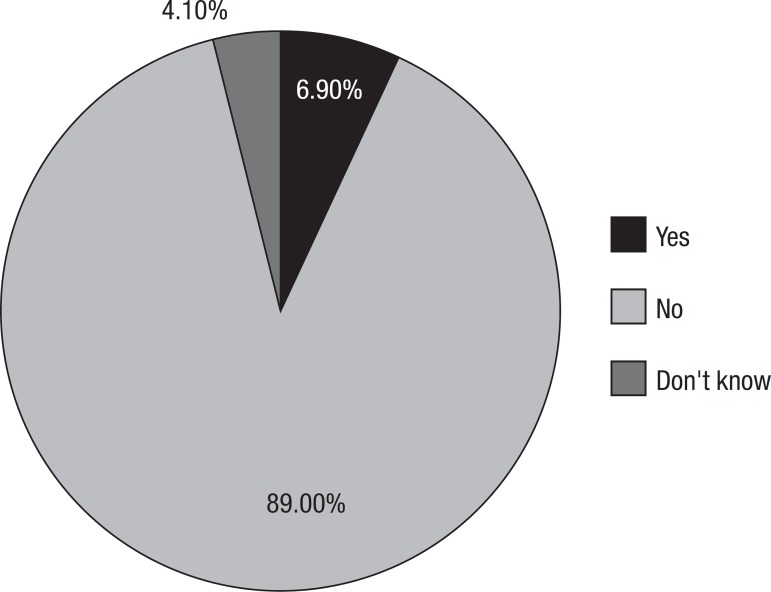
Can the demented stay at home alone?

When asked about changes to be expected from the elderly person (healthy old-aged), 56% of the interviewed cited behavioral/mood changes (anxiety, sadness, irritability, apathy, “to act like a child”), 23.3% stated memory impairment and 17.8% “movement disorder” (walks slowly, “loses agility”, falls) (Graph 3).

The interviewees used almost identical words to define the characteristics of demented individuals, although the proportion of answers in the 3 groups differed slightly: 41% cited memory impairment, 32.9% behavioral/mood changes and 8.2% “movement disorder”, while 17.8% gave no opinion (Graph 3).

**Graph 4:**
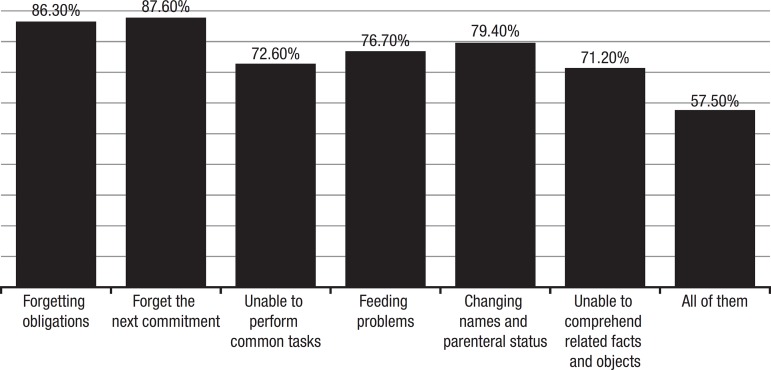
Behaviors associated with dementia

When asked if the demented can stay at home alone, 89% stated “no”, 6.85% said “yes” and 4% did not know (Graph 4).

**Graph 5:**
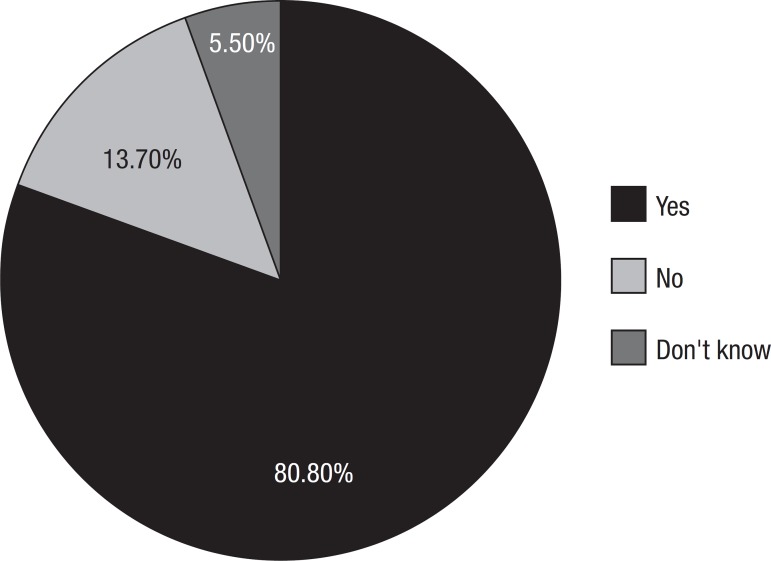
Do the demented present feeding difficulties?

A list of possible characteristics of healthy elderly and/or demented was presented (see questionnaire – Table 1) and 57.5% recognized all of the items as signs of dementia. Once again, there was a tendency to emphasize memory impairment (Graph 5).

**Graph 6:**
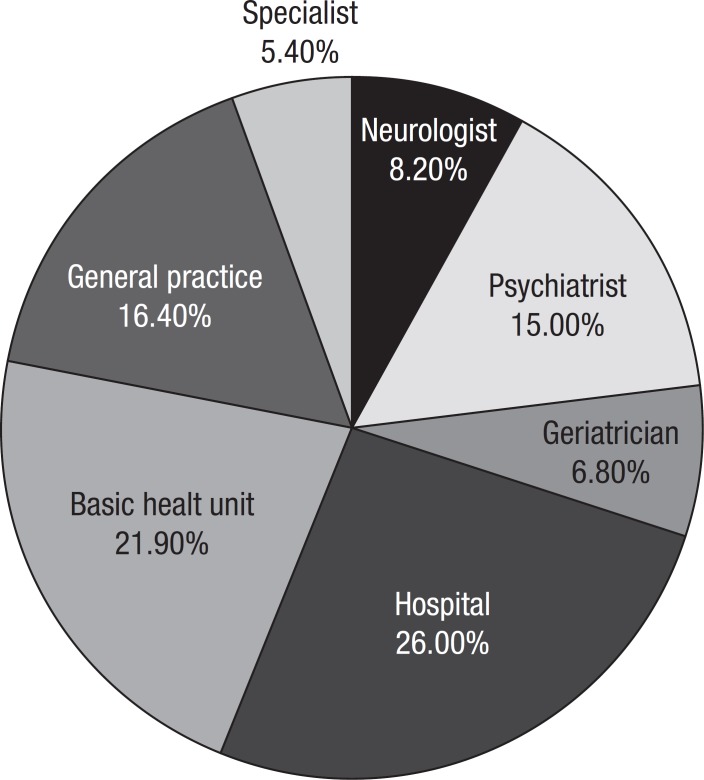
What kind of help would you look for if you suspected a relative had dementia?

Feeding difficulties in patients with dementia were also mentioned (80.8%). (Graph 6).

When asked how to proceed upon suspecting dementia, most interviewees would not seek specialized help where general hospital, general clinician, “doctor” and basic health unit combined accounted for almost 65% of the answers. “Neurologist” was cited by 8.2%, 15% psychiatrist, 6.85% geriatrician, and “specialized doctor” by 5.5%.

The wide range of responses and small size of our sample did not allow any complex statistical analysis.

## Discussion

Dementia is a chronic progressive illness that affects an increasing number of people worldwide. Estimates predict at least 71% of dementia patients will be living in the so-called developing countries by 2040.^4^ In 2005 a population study of dementia in a medium-sized city in São Paulo State registered a prevalence of 7.1%.^5^ Stenckenrider,^6^ in a community investigation in the United States of America, found that 91% had at least heard about Alzheimer’s disease, and while some had primary concepts from media, others had well founded scientific knowledge.

### Population sample characteristics

The studied adult samples have been from middle-middle class and low-middle class stratus. Several authors have sought to study the knowledge, attitudes and beliefs about dementia among a large sample of white, black and Hispanic adults.^7-8^ The registered differences do not apply to our population for several reasons. The first, concerns race and ethnicity of the Brazilian population, and the multiplicity of unspecific self appointed definition of race. Many considered to be black are recognized as “pardos”, some point between the black and white color spectrums. Other considered themselves “yellow” with no mention of origin, which may have been Asiatic, Indian (indigenous population) or in most case mixed race. Therefore, analysis of population awareness of Alzheimer disease or unspecific dementia among Brazilian samples of adults should focus on cultural and economic rather than Ethnic and racial aspects.

### Age of onset

Most of the answers given about age of onset of dementia were older ages, but 23.3% believed dementia would appear between 40 and 59 years’ old. This impression could be explained by the general notion of pre-senile dementia being the same as Alzheimer disease.

### Symptoms associated with dementias and the elderly

Notably, only 23.3% cited memory impairment in contrast to 56% stating behavioral changes as the main dysfunction among the elderly. However, when asked about symptoms among the demented, 41% cited memory disturbances. Barnes and al, in a study on the relationship between Alzheimer Disease, pathology and memory complaints, found that memory complaints were associated with AD pathology, both with and without clinically diagnosed AD. Interestingly, the association cannot be explained by depressive symptomatology or chronic health condition.^9^ It is important to remember that the present study refers to public opinion, and not to self-awareness of the degenerative pathologic process.

### Behavioral manifestations in elderly and demented patients

“Behavioral /mood changes” were believed to be present in the elderly by 56% of those interviewed while only 32.9% indicated that behavioral problems were associated with dementia. These results demonstrate that, in contrast to symptoms of memory impairment which were highly correlated with dementia, behavioral changes were not associated with dementia. Behavioral manifestations were apparently associated with psychiatric diseases but not necessarily with dementia. This issue was not however a subject of investigation in the present study.

It has been estimated that behavioral disturbances are present in 50% of Alzheimer patients.^10^ In a community study, a prevalence of behavioral problems (psychosis, depression, agitation) of 47% was established among subjects classified with mild cognitive impairment versus 66.1% of those diagnosed with dementia.^11^ In our population only 32.9% correlated dementia with behavioral symptoms, where the belief that behavioral manifestations are not associated with dementia prevailed. The registered answers are somewhat analogous in order to those found by Cruz et al.,^12^ in a study on family perception of initial cognitive and behavioral symptoms in Alzheimer patients. Memory was cited as the most recognized symptom by 93% and humor/mood changes by 80%.

One of the expressions used by the interviewees to describe behavioral change was “act like a child”, which could be understood as disinhibition, where this had an estimated prevalence of 11%.^13^

### Referral in case of suspected dementia

The fact that most of the interviewees would seek assistance from a general hospital, general clinician or basic health unit is in agreement with the recent implementation of family health programs that focus on families in their social environment.^14^ This also highlights the need for well trained professionals within basic health services. These healthcare professionals should be capable of identifying suspected dementia in order to achieve early diagnosis and better treatment.

### Movement disorders in the elderly and demented

The low percentage of people identified movement dysfunction as a symptom of aging or dementia indicates that slow walking, lack of agility and frequent falls are not frequently associated with age or dementia.

Cohen,^15^ analyzing the elderly population in India, points to the need of inclusion of socio and cultural aspects to evaluate the characteristics of senility and dementia. His findings suggest the need for an “anthropology of senility”.

The Brazilian population presents religious, cultural and ethnical diversity that could influence results, based on expectations from the healthy elderly population. Vernooij-Dassen and cols.^16^ studied the factors affecting timely recognition of dementia and concluded that more than specialist services are necessary to overcome delayed diagnosis. Stigma, social and family structure should also be addressed.^17-18^

Analysis of the overall findings of the present survey reveal that dementia was characterized by prevalent memory impairment (41%) and behavioral changes (32.9%) which begin in the 60’s or older (42.5%). On the other hand, senility was defined by behavioral/mood changes (56%) and memory impairment (23.3%). This is in agreement with Cohen’s findings in India: “anger rather than memory as a fundamental index of senile difference”.^15^

Although there are evident limitations to the application of the conclusions of this study to other population samples, our results demonstrate that the general clinician or basic health care providers are probably the first to evaluate the demented patients and to reach an early diagnosis.

An understanding of specific community awareness of dementia can promote more effective public health and educational policies. This can prevent delayed diagnosis and provide patient, family and potential caregivers with a better understanding of the disease course, thereby avoiding false expectations and decreasing psychological distress.

## Figures and Tables

**Graph 1:**
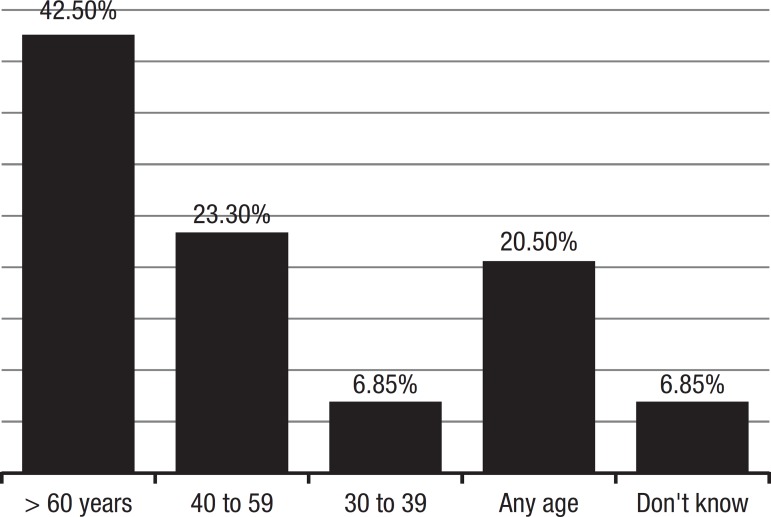
Expected age of dementia onset
